# N Partitioning between Gluten Fractions in a Set of Italian Durum Wheat Cultivars: Role of the Grain N Content

**DOI:** 10.3390/foods9111684

**Published:** 2020-11-18

**Authors:** Marina Mefleh, Rosella Motzo, Marie-Franҫoise Samson, Marie-Hélène Morel, Francesco Giunta

**Affiliations:** 1 Dipartimento di Agraria, Sezione Agronomia, Coltivazioni erbacee e Genetica, Università degli Studi di Sassari, Via De Nicola, 07100 Sassari, Italy; mmefleh@uniss.it (M.M.); giunta@uniss.it (F.G.); 2 IATE, Univ Montpellier, CIRAD, INRAE, Institut Agro, 34060 Montpellier, France; marie-francoise.samson@inrae.fr (M.-F.S.); marie-helene.morel@inrae.fr (M.-H.M.)

**Keywords:** durum wheat, grain N partitioning, grain N content, gliadin/glutenin, S-rich/S-poor

## Abstract

Grain protein content constitutes a key quality trait for durum wheat end-products and may also impact grain protein composition. A total of sixteen durum wheat cultivars were analyzed in a field trial during two seasons at two nitrogen (N) levels to evaluate whether and to what extent the variation in total grain N was associated with variation in the quantity of the various protein fractions and grain quality parameters. Genotypic variation in grain N content correlated with the variation in the content of all three protein fractions, although the strength of the correlation with gliadin and albumin-globulin was higher than that with glutenins. Genotypic variation in gliadin and glutenin content was more tightly correlated with the variation in the sulfur (S)-rich protein groups than with the S-poor protein groups and subunits. The variation in the percentage of unextractable polymeric proteins (UPP%) among genotypes was independent of their glutenin allelic composition. The significant genotypic differences in UPP% and in the ratios between protein groups and subunits were not influenced by the corresponding variation in grain N content. The final grain N content can only account for part of the variation in quality parameters and in the partitioning of total grain N between protein fractions since genotypic differences other than grain N content also contribute to these variations.

## 1. Introduction

Durum wheat (*Triticum turgidum* L. spp. *durum* Desf.) is mainly cultivated in the Mediterranean region, where it is primarily used as the preferred ingredient to make pasta, as well as a number of traditional and specialty breads [1]. A grain with a minimum protein content of 12–15% is required to manufacture pasta as it produces a uniform particle sized semolina, an elastic resilient non-sticky dough, and an al dente texture of the cooked pasta [2,3]. However, when the overall protein content is low to intermediate, a strong and tenacious gluten becomes necessary. Wheat gluten proteins endow wheat with its unique properties that are exploited in pasta and bread making. They form up to 80% of the total protein in grain, being accumulated and stored in the grain’s starchy endosperm. The mixing of durum wheat semolina with water leads to the formation of a viscoelastic network of gluten proteins, which endows the dough with its essential rheological properties [4,5]. Gluten proteins are composed of monomeric gliadins, which are categorized into the following groups: alpha/beta, omega, and gamma gliadins, and the polymeric glutenins, which comprise high molecular weight (HMW) and low molecular weight (LMW) glutenin subunits (GS) [4]. These protein subunits can also be categorized as either sulfur (S)-poor (HMW-GS and omega gliadins) or S-rich (LMW-GS and alpha/beta and gamma gliadins) [6], a classification which highlights the fundamental role of di-sulfide bonds for the formation of high molecular mass polymers [7]. Total protein content, the amount and ratios of the various storage proteins (mainly gliadins/glutenins, HMW/LMW-GS, and S-rich/S-poor proteins), and the percentage of grain unextractable polymeric proteins (UPP%, the proportion of large sodium dodecyl sulfate insoluble polymeric proteins) partly determine the levels of dough strength, elasticity, extensibility, and viscosity, all of which contribute to the biophysical and functional properties of dough and pasta quality [5].

The total grain protein content is not independent of the amounts and ratios of the various storage proteins. Indeed, although the overall partitioning of grain N between gliadins and glutenins in bread wheat at maturity has been demonstrated to be unmodified by growing conditions, it was found to vary according to differences in the total quantity of N accumulated in the grain [8,9]. However, these conclusions were reached by considering the pattern of accumulation of the various protein fractions during grain development for just one or very few bread wheat cultivars under different thermal and nutritional conditions. Hence, Triboi et al. [8] referred to the observed variation in grain N content as a consequence of grain growth, but they also implied that the protein composition of the grains of some bread wheat genotypes at harvest can simply be deduced by the total quantity of N per grain. At the same time, in the case of cultivars with different genotypes at the loci regulating the synthesis of different gluten fractions, quantitative ratios between fractions may differ despite the same total grain N content. In the case of Italian durum wheat cultivars, for example, a higher glutenin to gliadin ratio has been observed in modern compared with old cultivars, deriving from the expression of B-type LMW-GS, which was on average two times higher in the modern than in the old cultivars [10]. The importance of transcriptional control on the accumulation of gliadins and glutenins has also been noticed in bread wheat, and several putative loci identified that might regulate the differential expression of the different grain storage proteins [11].

Information on the existence of such relationships between total grain N and protein components and on the extent of environmental and genetic deviations from grain N allocation relationships is lacking with respect to durum wheat. To fill this gap, we analyzed the protein content and composition of a set of 16 durum wheat cultivars from different eras of breeding, hypothesized to retain a high level of genotypic variation in relation to grain protein content and composition. The set specifically contained a large number of old cultivars since the level of genetic variability in gluten characteristics present in old durum wheat cultivars from the Mediterranean basin is known to be much larger than that of modern cultivars [12]. At the same time, substantial differences exist in protein content and in grain quality traits between old and modern cultivars, since modern cultivars generally have lower protein percentages [13,14] but stronger gluten as a consequence of the introgression of more favorable alleles for both HMW and LMW-GS. The aim of our study was to find out whether, and to what extent, the genotypic variation in total grain N at maturity is also responsible for variations in the amounts and proportions of the different grain protein fractions and subunits analyzed.

## 2. Materials and Methods

### 2.1. Site, Treatments, and Experimental Design

The experiment was carried out during seasons 2015/16 (‘2016’) and 2016/17 (‘2017’) at the experimental station belonging to the University of Sassari, located in Ottava (41° N; 8° E; 80 m above sea level). The location is a typically Mediterranean environment, with a long-term mean annual rainfall of 539 mm—the majority of which occurs between October and April.

A total of 16 cultivars of durum wheat (*Triticum turgidum* L. ssp. *durum* Desf.), from different eras of wheat breeding and with different gluten allelic composition, were compared (Table 1). Most of them were tall cultivars, which were diffuse across Italy in the first half of the 20th century. Two cultivars, Ichnusa and Maristella—deriving from crosses between *mediterraneum* and *syriacum* types [15], and widely grown in the 1960s—are generally shorter and earlier than the old cultivars, but still prone to lodging. Two semi-dwarf varieties, Svevo and Aureo, were also investigated, which were chosen from the many available semi-dwarf Italian durum wheat cultivars because of their reputation for producing good quality pasta [10].

Cultivars were sown on 10 December 2015 (2016 season), and 15 November 2016 (2017 season), at a sowing rate of 250 viable seeds m^−2^, and at two fertilization rates: a low N level (‘N46’) with a single nitrogen application at the time of sowing (46 kg ha^−1^ in the form of urea); a high N level (‘N86’), which benefited from a second application of 40 kg ha^−1^ at the onset of stem elongation in the form of ammonium nitrate.

The soils, just 0.4–0.5 m deep due to an underlying layer of limestone, have an organic matter content of 1.4 ± 0.3%, 45 kg ha^−1^ of mineral nitrogen, 8.4 ± 0.5 ppm of available phosphorus and high total CaCO_3_ (40 ± 4.4%). The available water amounted to 61 mm. In both seasons, the preceding crop was faba bean, and the sowing bed was prepared by ploughing to a depth of 0.25 m followed by surface cultivation. Weeds, pests, and diseases were chemically controlled.

Each plot consisted of six 1.2 m rows with an inter-row distance of 0.18 m. Plots were separated from each other by 50 cm all around to ensure the absence of interference between neighboring plots. The plots were set out as a split-plot design with three replications. Cultivars were assigned to the main plots and nitrogen rates to the sub-plots.

### 2.2. Measurements and Analysis

Anthesis date and physiological maturity were recorded when 50% of plants had reached these phenological stages, as ascertained from periodical inspections of the plots. Grain filling duration was estimated as the difference between maturity and anthesis.

Grain moisture content and grain weight were obtained from four 250 grain sub-samples per plot. Nitrogen percentage was determined on each sub-sample by means of a Carbon/Hydrogen/Nitrogen Analyzer (628 Series, LECO Corporation, St. Joseph, MI, USA). Nitrogen data were used to calculate grain protein percentage, on a dry basis, as N percentage × 5.7 and the amount of nitrogen (in µg) per grain (total grain N) as grain weight at dry basis × N percentage.

#### 2.2.1. Sequential Extraction of Gliadin and Glutenin for Reverse Phase-High Performance Liquid Chromatography (RP-HPLC) Analysis

Albumins-globulins, gliadins and glutenins were sequentially extracted from 50 mg of ground wholemeal grain as described by Wieser and Seilmeier [16] and separated at 50 °C on an ACE C18 column (250 × 2.1 mm, 5 µm, 300 Å) using an Alliance HPLC system (Waters). The elution system consisted of (A) trifluoroacetic acid (TFA, 0.1%, *v*/*v*) and (B) acetonitrile/TFA (99.9/0.1%, *v*/*v*). Linear gradient: 0 min 20% B, 20 min 60% B (albumins-globulins); 0 min 28% B, 30 min 56% B (gliadins, glutenin subunits). Flow rate was 0.2 mL min^−1^; detection was UV absorbance at 210 nm. Following RP-HPLC separation, the area under the protein curve was calculated, corresponding to the amount of protein extracted in each fraction. For each grain sample, the protein extracted was then expressed as an amount per grain. Gluten amount was obtained by summing the gliadin and glutenin peaks. Total protein content was estimated by adding the albumin-globulin, gliadin, and glutenin peaks together. Areas corresponding to gliadin groups (alpha/beta, omega, and gamma) were calculated by cutting the whole gliadin chromatogram up as described by Samson et al. [17] with some adjustments: 7 to 14 min (omega); 14 to 22 min (alpha/beta); 22 min until the last peak (gamma). The same procedure was applied to glutenin chromatograms: 7 to 17 min (HMW-GS); 17 min to the last glutenin peak (LMW-GS) [18].

The relative percentage of each protein fraction (albumin-globulin, gliadin, and glutenin) was calculated from total protein, and the relative percentage of each gliadin group (i.e., alpha/beta, omega, and gamma) and the glutenin subunits (HMW and LMW) was calculated from total gluten.

Gliadin over glutenin ratio was calculated as the ratio of the gliadin peak over glutenin peak and the S-rich over S- poor ratio was calculated as the ratio of the sum of alpha/beta, gamma, and LMW-GS peaks over the sum of omega and HMW-GS peaks.

#### 2.2.2. Extraction of Extractable and Unextractable Polymeric Proteins for Size Exclusion-High Performance Liquid Chromatography (SE-HPLC) Analysis

Extraction of polymeric protein and separation by SE-HPLC was conducted as described by Dachkevitch et al. [19] and Morel et al. [20] with minor modifications. Briefly, SDS-soluble proteins were extracted from 160 mg wholemeal flour by the addition of 20 mL 1% (*w*/*v*) SDS in 0.1 M sodium phosphate buffer (pH 6.9) and incubated on a rotary shaker (60 rpm at 60 °C for 80 min). Following centrifugation (37,000× *g*, 30 min, 20 °C), the supernatant was set aside for further SE-HPLC analysis. The unextractable proteins (or SDS insoluble proteins) were obtained by adding 5 mL SDS-phosphate buffer to the pellet, vortexing, and sonicating for 3 min at 7.5 watts, then centrifuging at 37,000× *g* at 20 °C for 30 min. The supernatant was subjected to SE-HPLC analysis and the pellet discarded. SE-HPLC separations were performed on an Alliance system (Waters) equipped with a TOSOBIOSCIENCE TSKgel G4000SWXL column (8 × 300 mm, 8 µm, 300 Å) protected by a guard column TOSOBIOSCIENCE TSKgel SWXL guard (6 × 40 mm, 7 µm mm). Proteins were eluted at ambient temperature with 0.1 M sodium phosphate buffer (pH 6.9) containing 0.1% (*w*/*v*) SDS at a constant flow of 0.7 mL min^−1^ and absorbance was recorded at 214 nm.

The total area under each chromatogram obtained from SDS extractable and unextractable protein extracts was expressed as a percentage (EP% and UP%, respectively) of the sum of the total area of both chromatograms. The unextractable polymeric protein fraction (UPP%) was calculated as a percentage of the total polymeric proteins (UPP% = UP%/(sum of glutenins % of EP) + UP%).

Grain nitrogen data and the percentages of protein fractions calculated by RP-HPLC were used to estimate the µg of nitrogen of each protein fraction, considering that we can express proteins in terms of nitrogen and vice versa (grain protein = grain N × 5.7) and that the proportion of non-protein nitrogen in wheat grain at maturity and in the semolina is very low to negligible [21,22,23]. The nitrogen contents of EP and UP were calculated using their percentages (calculated by the SE-HPLC) multiplied by grain nitrogen (total grain N). The nitrogen contents of albumin-globulins, gliadins, and glutenins were calculated using their percentages calculated by RP-HPLC multiplied by the nitrogen content of EP. The nitrogen contents of the gliadin groups and the glutenin subunits (alpha/beta, omega, and gamma and HMW and LMW-GS) were calculated using their percentages calculated by RP-HPLC multiplied by the nitrogen content of gluten. From here on, the nitrogen content of a fraction or subunit will simply be referred to as ‘content’; for example, the µg of nitrogen of gliadin will be referred to as ‘gliadin content’.

#### 2.2.3. Electrophoresis

Gliadins were analyzed by A-PAGE, and the gliadin extraction was carried out on 30 mg durum wheat flour as described by Morel [24]. HMW-GS were separated by SDS-PAGE, and protein extraction was performed on 20 mg durum wheat flour as described by Singh et al. [25]. The identification of high-molecular-weight glutenin subunit (HMW-GS) alleles was based on the classification proposed by Payne and Lawrence [26].

### 2.3. Statistical Analysis

Agronomic data were analyzed by combined ANOVA, using R software [27], according to a split-split plot design with three replications, where years were assigned to the main plot, cultivars to the sub plots, and nitrogen treatment to the sub-sub plots. Cultivar means were separated by means of a multiple *t*-test once the level of statistical significance of the cultivar effect had been ascertained. The Pearson correlation coefficient was used to evaluate the existence of causal relationships between pairs of traits.

## 3. Results

### 3.1. Weather Conditions and Phenology

**Figure 1:**
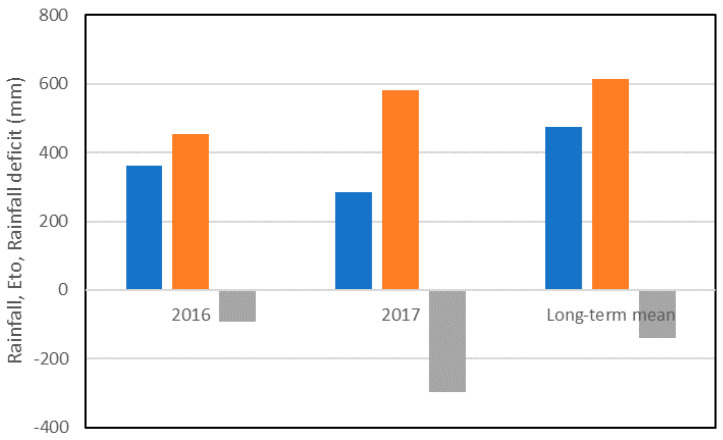
Rainfall (blue bars), reference evapotranspiration (ETo) (orange bars), and rainfall deficit (rainfall–ETo) (grey bars) between October and May for the years 2016 and 2017, plus the long-term mean (40 years; 1970–2010).

The rainfall recorded between October and May amounted to 286 mm in 2017 and to 363 mm in 2016, i.e., 60% and 76%, respectively, of the long-term mean (40 years) (Figure 1).

Rainfall was particularly scarce in the springs of both seasons; a total of only 8 mm of rain fell in April and May in 2017, and 25 mm in 2016. 2017 was also characterized by higher maximum temperatures in May (24.1 °C compared with 21.2 °C in 2016), when most of the grain filling occurs, as well as by a greater number of days in which the maximum temperature exceeded 25 °C (17 vs. 4 days in 2016).

Phenology was not affected by the N treatment, whereas the effect of Cultivar and Cultivar × Year interaction were both significant as shown by ANOVA (Table 2).

In relation to anthesis date, the cultivars could be divided into two groups: (i) those with a mean anthesis date that fell within the first week of May (in the 2017 season, this resulted in less than one month for grain filling in the case of some cultivars), which included almost all the tall and old cultivars; (ii) those with a mean anthesis date between April 17 and 20, which enabled a longer grain filling period compared with the first group, and comprised the four more recent constitutions. These differences in anthesis date resulted in different thermal conditions during grain filling: the first group of cultivars (made up of the old, later varieties) was exposed to higher maximum temperatures compared with the earlier flowering group (24.5 vs. 22.3 °C in 2017, and 21.7 vs. 20.4 °C in 2016) and had more days in which the maximum temperature exceeded 25 °C, especially in 2017 (14 vs. 10 days in 2016).

### 3.2. Characterization of Gliadin and Glutenin Alleles

Based on their mobility on the acid and SDS-PAGE, six HMW-GS patterns were revealed: 20, 20*, 7, 13 + 16, 6 + 8, and 7 + 8. Particularly, one variety, Saragolla, produced a heterogeneous HMW-GS pattern (6 + 8; 20) that was different to the one reported by De Santis et al. [10] This is probably because landraces tend to be mixtures of different pure lines. All the genotypes studied had the superior LMW2 associated with gliadin *γ*-45, except for Saragolla, which had the unusual combination of LMW2/gliadin *γ*-42 (Table 1).

### 3.3. Grain Nitrogen and Its Partitioning

No effect of year was detected on the nitrogen content of the grain in spite of the more severe terminal drought and higher temperatures characterizing the grain filling period in 2017, whereas both grain nitrogen content and protein percentage increased at the higher N rate (Table 3).

The ranking of the cultivars in relation to the content and percentage of N in the grains was not influenced by the year, nor by the nitrogen treatment, as demonstrated by the lack of any significant interaction of N and/or year with cultivar. The genotypic variation expressed by the 16 cultivars on the average of the two years and two N treatments was large for both grain protein percentage (12.4–17.3%) and total grain N content (735–1453 µg N grain^−1^), and was mirrored in a significant effect of cultivar for all the traits presented in Table 3. The differences in grain protein percentage between the cultivars was derived from the corresponding differences in grain N content, the two being associated traits, with an *r* = 0.81 ***.

A lower gliadin content was observed at the higher N rate (N86) (Table 3) due to a lower gamma gliadin content, while alfa/beta and omega groups remained unchanged (Table 4).

However, this negative effect of the fertilization treatment on gliadins was only true in 2016 (464 µg grain^−1^ at N46 vs. 302 µg grain^−1^ at N86), whereas the opposite was true in the drier 2017 season. In 2017, the gliadin content at N86 was 14.6% higher than that at N46 (400 µg grain^−1^ at N46 vs. 471 µg grain^−1^ at N86) for all cultivars except for Trigu biancu, Trigu murru, and Trigu arrubiu. Consequently, significant N × Year and N × C interactions were generated (Table 3).

The glutenin plus albumin-globulin content was higher at N86 than at N46 (12% and 10%, respectively). However, the glutenin contents of Cappelli and Dauno were negatively affected by the additional N fertilization in both years, generating an N × C interaction (Table 3). Both glutenin subunits (HMW and LMW) varied, by the same degree, with N fertilization (Table 4). The described variation in gliadins and glutenins caused the gliadins to glutenins ratio (GLI/GLU) to be higher at N46 (1.04) than at N86 (0.84) (Table 3). The gliadins constituted the only protein fraction to be affected by year, with a higher gliadin content being observed in 2017—the year characterized by severe water and temperature stress (437 vs. 383 µg grain^−1^ in 2016). The content of the different gliadin groups increased accordingly, with the exception of omega gliadins. As a consequence, GLI/GLU was higher in 2017. UPP% was lower at N86 and in 2017—the drier and hotter year. The negative effect of an additional N application on UPP% was stronger in 2017 than in 2016 for all the cultivars studied.

**Figure 2:**
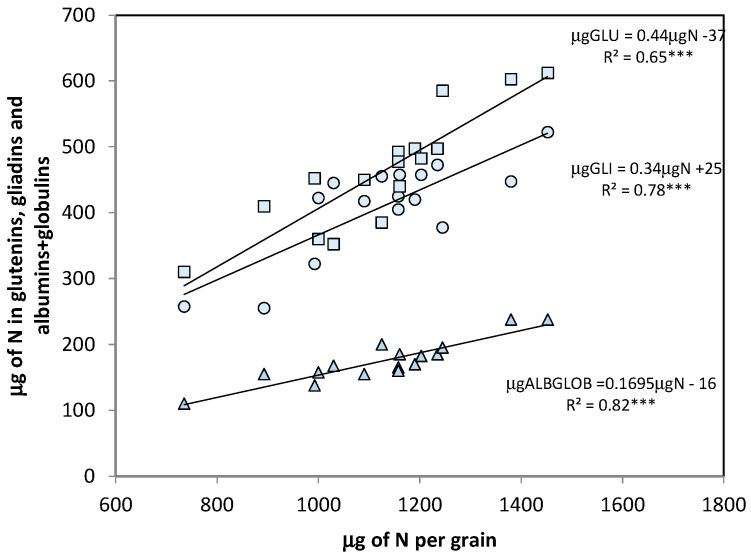
Relationships between the variation in the total grain nitrogen and the N content of the three protein fractions: glutenins (GLU) (squares), gliadins (GLI) (circles), and albumins-globulins (ALBGLOB) (triangles). Points are cultivar means across years and N treatments. *** *p* ≤ 0.001.

To evaluate whether the genotypic variation in total grain N also implied a variation in the various protein fractions, cultivar means were used to calculate the relationships between albumin-globulin, gliadins, and glutenins with total N content. Given the significant cultivar × N interaction for gliadins and glutenins, as shown by ANOVA (Table 3), we first regressed the two N treatments separately, but because their slopes and intercepts were not statistically different, only the results for a regression based on cultivar means across year and N treatments are discussed (Figure 2).

These relationships provided information about the strength of the relationship between genotypic variation in protein fractions and the total grain N (coefficient of correlation and determination), and the extent of the variation in protein fractions for each unit of variation of total grain N, or partitioning coefficient (slope of the regression).

The albumins-globulins protein fraction was the one most tightly associated with total grain N, which accounted for 82% of its genotypic variation, compared with 78% for gliadins and 65% for glutenins. On the other hand, the albumins-globulins fraction was also the one with the lowest slope of regression (0.17 ± 0.02) compared with the storage proteins, gliadins (0.34 ± 0.07), and glutenins (0.44 ± 0.06); i.e., it was the fraction that varied the least in response to the genotypic variation in total grain N. The relationship between total grain N and gliadin was stronger than that between total grain N and glutenins, and no significant differences were detected between either the slopes of these two relationships, or between their intercepts, which were not different from zero. A significant effect of cultivars on the GLI/GLU ratio was detected by ANOVA, reflecting the variation in GLI/GLU from 0.63 to 1.35 (Table 3), but this variation in GLI/GLU was not associated with the variation in total grain N (data not shown).

**Figure 3:**
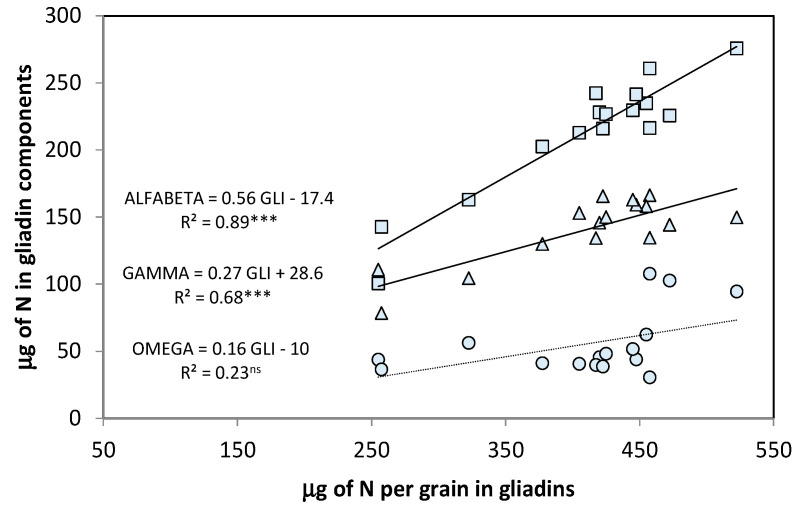
Relationships between the variation in the µg of total gliadins per grain and the content of the three gliadin components: alpha/beta (squares), gamma (triangles), and omega (circles). Points are cultivars means across years and N treatments. ***, *p* ≤ 0.001; ns, not significant.

Within the gliadin groups, the sulfur(S)-rich alpha/beta and gamma were generally present in larger amounts (101–261 µg for the alpha/beta, and 79–166 µg for the gamma gliadins), than the S-poor omega gliadins (31–108 µg) (Figure 3). The relationship between total gliadin content and alpha/beta gliadins was stronger (*R*^2^ = 0.89 ***) than the relationship with gamma gliadins (*R*^2^ = 0.68 ***), whereas omega gliadin levels varied independently from the total gliadin content, in spite of the large genotypic variation in this component. The slope of regression of alpha/beta (0.56 ± 0.05) was significantly different and two times higher than that of gamma (0.27 ± 0.05).

**Figure 4:**
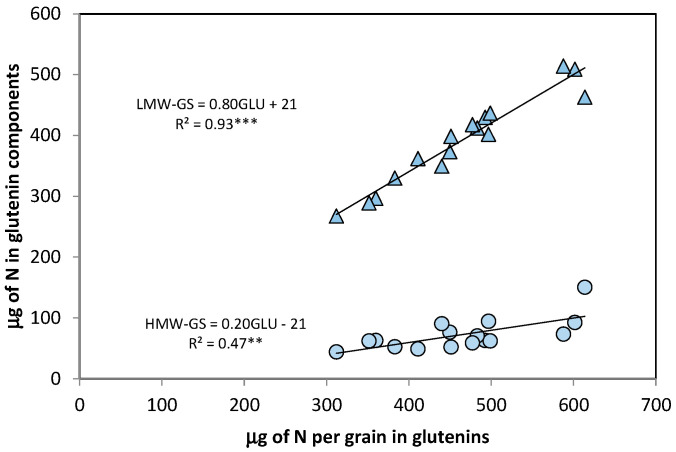
Relationships between the variation in the µg of total glutenins per grain and the amount of the two glutenin components: LMW-GS (triangles) and HMW-GS (circles). Points are cultivar means across years and N treatments. *** *p* < 0.001, ** *p* < 0.01.

The S-rich LMW-GS were present in larger amounts than the S-poor HMW-GS (Figure 4) and showed greater genotypic variation: 270–510 µg grain^−1^ (coefficient of variation = 36 vs. 19% for HMW-GS). The genotypic variation in grain N allocated to glutenins was more tightly associated with the variation in LMW-GS (*R*^2^ = 0.93 ***) than in HMW-GS (*R*^2^ = 0.47 **).

The slope of regression of the content of LMW-GS per grain (0.80 ± 0.06) was more than four times greater than that of the content of HMW-GS (0.20 ± 0.06). In spite of these linear relationships, the HMW/LMW ratio was significantly different between genotypes as shown by ANOVA, ranging from 0.13 to 0.33 (Table 3), but no relationship was found between that ratio and total N grain glutenin content.

The S-rich to S-poor ratio did not vary with total grain N, but it was significantly different among genotypes and varied from 3.7 to 8.3 (Table 3). Similarly, UPP% did not vary with total grain N, although it was negatively correlated with HMW/LMW (*r* = 0.88 ***) and positively correlated with S-rich/S-poor (*r* = 0.93 ***).

## 4. Discussion

Grain protein percentage makes the largest contribution (40%) to the EU Quality index for durum wheat (European Commission Regulation No. 2237/2003, 23 December 2003), and in some countries, grain protein concentration influences the amount of money paid to wheat farmers due to the importance of this trait for the quality of both pasta and bread. In bread wheat, a higher protein content is generally equated with higher protein quality for bread making [28], consistent with the association between grain N content and protein fraction composition, as first highlighted by Triboi et al. [29] and then used by Martre et al. [30] to simulate grain N composition in the Sirius Quality model. An important finding obtained from this approach was that the water availability, the temperature during grain filling, and the nitrogen fertilization rate can only affect protein composition through a variation in total grain N. All of these relationships were investigated in the present paper in durum wheat, including testing for the effect of genotype, because not only did we expect genotype to affect the proportions of gluten protein fractions and components, we also expected it to interact with the environment.

### 4.1. Nitrogen and Season Effect

The effect of N fertilization on grain protein fractions across seasons and genotypes was mediated by an effect on the total grain N, as previously suggested by Triboi et al. [8], and consisted in a change in the GLI/GLU ratio, as glutenins increased and the gliadin fraction decreased. This result lies in contrast with some previous studies on bread wheat [31,32,33]. The significant Cultivar × N and N × Year interactions for gliadin and glutenin content in our study suggest that the effect of N fertilization on grain protein is strongly dependent on the specific cultivar considered, in agreement with Pechanek et al. [34] and Wieser and Seilmeier [16], and on the environmental conditions under which it is grown.

N fertilization also affected the quality parameters S-rich/S-poor and UPP%. Our results agree with those of Johansson et al. [35,36], who showed that N fertilization decreases the UPP%, and with other authors who found that N supply leads to a lower S-rich and higher S-poor protein component [37,38]. In bread wheat grown under controlled conditions, this change in S-rich/S-poor ratio was not due to a S deficiency, and the addition of post-anthesis S had no effect on protein composition [36]. On the other hand, our results are in contrast with those of Gagliardi et al. [39], who showed that N supply increases UPP% in durum wheat.

We found a similar total grain N and grain protein percentage between the two years in spite of the more severe stress conditions during grain filling and the shorter grain filling duration encountered in 2017. This shorter grain filling was the likely cause of the lower UPP% observed in 2017 since the accumulation of SDS-insoluble large polymer proteins in the grain increases in the final stages of grain growth and is related to continuous grain dehydration [40]. The only protein fraction affected by year was the gliadins—as also noted by Daniel and Triboi [41]—specifically the S-rich gliadin groups, which led to an increase in the S-rich/S-poor ratio. However, the interactions between year and N supply in grain protein components and the UPP% make the interpretation of the effect of each factor alone complicated, as also noted by Johansson et al. [36] and Malik et al. [42]. On the other hand, no Cultivar × Year interaction was observed for any of the protein fractions considered in spite of the different environmental conditions experienced by the set of cultivars analyzed as a consequence of their different anthesis dates.

### 4.2. Genotypic Effect

The variation in grain protein percentage and absolute content was considerable for the set of cultivars investigated. This allowed us to assess the dependence of genotypic variation in grain protein percentage on the variation in total grain N, and to describe the relationships between total grain N and protein fractions for durum wheat. By expressing nitrogen on a per grain basis in µg rather than as a percentage, we were also able to exclude the confounding effect of the variation in carbohydrate accumulation–the processes of N and carbohydrate accumulation being largely independent of each other [43].

As total grain N varied among genotypes, the variation in the content of the storage proteins exceeded that of the metabolic proteins; i.e., each µg of total grain N difference between cultivars was associated with an increase in the µg of N in gliadins and glutenins, and this increase was more than twice the increase in µg of N observed in albumins-globulins. Indeed, metabolic protein accumulation is known to be sink limited [9,30], in contrast with storage proteins which are source-limited.

The genotypic variation in total grain N was more tightly associated with the variation in gliadins than in glutenins, at least in the set of cultivars and environmental conditions analyzed here. This meant that genotypic deviations for glutenins were large, a result that can be explained by the presence of polymorphisms in the HMW-B1 glutenin subunits (six different HMW-B1 types among our 16 cultivars), which also implied differential expression levels of the specific storage proteins [10].

Interestingly, the partitioning coefficients calculated in this experiment between N in gliadins and total grain N (0.34 ± 0.07), and N in glutenins and total grain N (0.44 ± 0.06), were similar to those calculated by Triboi et al. [8] for the second phase of the grain filling period of bread wheat (0.34 ± 0.01 for gliadins and 0.46 ± 0.01 for glutenins). This means that the relationships described by Triboi et al. [8] between the storage protein fractions and total N of developing grains of bread wheat grown under varying environmental conditions (post-anthesis temperature and water availability, and rate and timing of N fertilization) also hold for mature grains of durum wheat when the variation is induced by genotypic differences.

The proportions of the different gliadin groups in the total gliadins were the highest for alpha/beta compared to gamma or omega, which is in accordance with the literature on bread wheat [29,34,44], and the proportion of glutenin subunits in total glutenins was higher for LMW than HMW-GS, as already found in bread wheat [29].

The genotypic variation in both gliadins and glutenins was more strongly associated with their respective S-rich components (alfa-beta gliadins gamma gliadins, and LMW-GS) than with their S-poor components. The S-poor omega subunit did not vary with the variation in total µg of gliadins per grain, in accordance with Uthayakumaran et al. [45], and the coefficient of determination between total glutenins and LMW-GS was double the value obtained for HMW-GS. The S concentration of the grain is an important indicator of the size distribution of the gluten macropolymers that govern the rheological properties of dough [37,45,46,47]. Therefore, it is reasonable to suppose that durum wheat cultivars with high total grain N and gluten content would also produce a more extensible dough due to their high content of S-rich proteins [6].

The significant genotypic differences in the GLI/GLU, S-rich/S-poor, and HMW/LMW ratios and in UPP% were not influenced by the corresponding variation in grain N content. In contrast with our results, Blumenthal et al. [48], Jia et al. [33], and Triboi et al. [29] found a positive relationship between GLI/GLU and grain N content, and Pechanek et al. [34] found a positive relationship between HMW/LMW and grain N content. In accordance with our results, Triboi et al. [29] did not identify any relationship between the HMW/LMW ratio and grain N content, and Zhang et al. [49] agree that UPP% largely depends on genotype. The reasons for this discrepancy could be a consequence of the different species analyzed (bread wheat was addressed in the cited papers, whereas durum wheat was the subject of the present study) and/or in the size of the sets of cultivars studied, which was smaller in most of the cited papers, whereas the present study included cultivars from different breeding eras, which therefore expressed large polymorphisms in their protein fractions.

According to Wrigley et al. [50], UPP% is greatly affected by the gluten allelic composition of the cultivars. However, our modern varieties, Aureo and Svevo, contain the improved versions of the glutenin HMW alleles at the Glu-B1 locus (6 + 8 and 7 + 8, respectively) and had high UPP% values that did not differ to those for Calabria and Cappelli, which have the HMW 20 genotype at the Glu- B1 locus that is considered weaker than both HMW 7 + 8 and 6 + 8 for producing good quality pasta and bread [51,52].

## 5. Conclusions

In the set of cultivars studied and environmental conditions explored, the linear relationships calculated between protein fractions and total grain N accounted for the majority of the variation in protein composition, but cultivar-dependent deviations with respect to these relationships were generally large enough as to significantly influence the ratios between fraction and, hence, presumably, gluten quality. The variation in protein fractions associated with a variation in total grain N content depends on the cause of the variation in µg N per grain. A greater N availability implies an increase in glutenin and a concomitant decrease in the average gliadin content, although this response was found to be genotype-dependent. On the contrary, when a cultivar-dependent increase in N content occurs, the relationship with total grain N is positive for both gliadins and glutenins, although the S-rich gluten components are more strongly associated with total grain N.

## Figures and Tables

**Table 1:** The investigated genotypes, their relative year of release, geographic or genetic origin, and details of the allelic composition of gluten genes.

Name	Geographic or Genetic Origin	Year of Release	Glu-A1	HMW-GS-B1	LMW-GS-B3	Gliadin-A1
Calabria	Calabria	-	Null	20	2	45
Dauno	Apulia	1914	Null	6 + 8	2	45
Russello	Selected from a Sicilian landrace	1936	Null	13 + 16	2	45
Saragolla ^(1)^	Apulia	-	Null	6 + 8; 20	2	42
Scorzonera	Indigenous landraces from Sicily	-	Null	20	2	45
Senatore Cappelli	Nord-african landrace Jean Retifah	1915	Null	20	2	45
Taganrog	Russia	-	Null	20*	2	45
Timilia	Sicily	-	2*	20	2	45
Trigu arrubiu	Sardinia	-	Null	20	2	45
Trigu biancu	Sardinia	-	Null	20	2	45
Trigu murru	Sardinia	-	2^+^	20*	2	45
Triminia	Sicily	-	2^+^	7	2	45
Ichnusa	Biancale × Capeiti 8	1968	Null	20	2	45
Maristella	Dauno-III × Capeiti-8	1968	Null	20	2	45
Svevo	Sel. CIMMYT × Zenit sib	1996	Null	7 + 8	2	45
Aureo	Kofa × Svevo	2009	Null	6 + 8	2	45

^(1)^ Saragolla seeds were heterogeneous regarding the HMW-GS pattern.

**Table 2:** Anthesis date and grain filling duration. Least Square Means and LSD_0.05_ for the comparison of cultivar × year means at the same or different levels of year.

Cultivar	Anthesis		Grain Filling Duration	
	(Doy) ^(1)^		(Day)	
	2016	2017	2016	2017
Calabria	124	121	32	30
Cappelli	125	122	31	30
Dauno	127	124	30	27
Russello	125	123	32	29
Saragolla	126	125	32	26
Scorzonera	128	127	29	25
Taganrog	126	124	31	27
Timilia	116	114	33	34
Trigu arrubiu	123	123	33	28
Trigu biancu	125	124	30	28
Trigu murru	123	123	32	28
Triminia	123	125	33	27
Ichnusa	109	109	39	38
Maristella	110	110	40	37
Svevo	107	110	40	35
Aureo	107	110	42	36
Mean	120	120	34	30
LSD_0.05_	2		2	

^(1)^ Doy: day of the year.

**Table 3:** Grain protein percentage, total grain N, N in gliadins, glutenins and albumins-globulins (μg of N in each component), the ratios gliadins to glutenins (GLI/GLU), high molecular to low molecular weight glutenins subunits (HMW/LMW) and sulfur rich to sulfur poor (Srich/Spoor), and unextractable polymeric proteins percentage (UPP%). Least Square Means and results of the analysis of variance.

	Grain Protein	Total N	N in Gliadins	N in Glutenins	N in ALBGLOB	GLI/GLU	HMW/LMW	Srich/Spoor	UPP%
	(%)	(µg grain^−1^)							
Year (Y)	ns	ns	**	ns	ns	**	ns	*	***
2016	14.6	1110	383 b	468	179	0.85 b	0.19	6.16 b	28.5 a
2017	14.8	1146	437 a	458	171	1.03 a	0.18	6.43 a	27.5 b
Nitrogen (N)	***	**	*	***	**	**	ns	***	***
N46	14.3 b	1093 b	433 a	434 b	166 b	1.04 a	0.18	6.62 a	28.4 a
N86	15.2 a	1138 a	387 b	492 a	184 a	0.84 b	0.19	5.98 b	27.6 b
Cultivar (C)	***	***	***	***	***	***	***	***	***
Calabria	14.3 df	1158 bc	405 bc	493 b	165 be	0.83 cf	0.15 gi	7.67 ab	30.2 ab
Cappelli	14.6 df	1190 bc	420 ac	498 b	170 be	0.85 cf	0.15 hi	7.52 bc	30.4 ab
Dauno	16.8 ab	1235 b	473 ab	498 b	185 bd	0.98 bd	0.24 c	3.91 h	25.5 e
Russello	15.0 ce	1090 ce	418 ac	450 bd	155 de	0.93 ce	0.20 d	6.45 eg	25.3 e
Saragolla	15.8 bc	1160 bc	458 ab	440 bd	185 bd	1.09 ac	0.26 b	3.53 h	25.7 e
Scorzonera	14.5 df	1000 df	423 ac	360 ef	158 ce	1.26 ab	0.21 d	6.67 ce	25.9 e
Taganrog	15.2 cd	1203 bc	458 ab	483 bc	183 bd	0.96 bd	0.17 ef	8.29 a	26.5 ce
Timilia	13.8 cd	1125 a	455 ab	385 de	200 ab	0.80 cf	0.18 e	6.27 cf	29.0 ac
Trigu arrubiu	14.8 cf	1245 b	378 bc	585 a	195 bc	0.65 ef	0.15 hi	7.39 bd	28.5 ab
Trigu biancu	15.2 f	1380 bd	448 ab	603 a	238 a	1.26 ab	0.16 fh	6.65 eg	28.1 bd
Trigu murru	17.3 a	1453 a	523 a	613 a	238 a	0.95 ce	0.33 a	3.62 h	23.7 e
Triminia	15.1 ce	1030 de	445 ab	353 ef	168 be	1.35 a	0.22 d	5.98 fg	26.5 de
Ichnusa	14.4 df	1158 bc	425 ac	478 bc	160 ce	0.91 cf	0.14 ij	7.39 bd	29.9 ab
Maristella	12.5 g	893 f	255 d	410 ce	155 de	0.63 f	0.14 ij	6.18 eg	30.9 a
Svevo	12.4 g	735 g	258 d	310 f	110 f	0.85 cf	0.16 fg	6.03 dg	28.8 ab
Aureo	14.0 ef	993 ef	323 cd	453 bd	138 ef	0.7 df	0.13 j	6.14 g	28.9 ab
Y × C	ns	ns	ns	ns	ns	ns	ns	ns	ns
N × C	ns	ns	*	*	ns	*	ns	*	ns
N × Y	***	***	***	ns	**	***	ns	ns	*

Level of significance: * *p* ≤ 0.05; ** *p* ≤ 0.01, *** *p* ≤ 0.001; ns: not significant. Means with the same letter are not statistically different as assessed using the Student Test for *p* ≤ 0.05.

**Table 4:** Grain content of the gliadin groups (alfa/beta, omega, and gamma), and of the glutenin subunits (high- and low-molecular weight: HMW and LMW) (µg of N in each component). Results from Least Square Means and the analysis of variance ANOVA.

	N in Alfa/Beta Gliadins	N in Omega Gliadins	N in Gamma Gliadins	N in HMW-GS	N in LMW-GS
	(µg)	(µg)	(µg)	(µg)	(µg)
Year (Y)	**	ns	**	ns	ns
2016	202	51.6	129	73.9	394
2017	225	58.9	152	70.8	388
Nitrogen (N)	ns	ns	*	***	***
46	222	55.1	156	66.3	368
86	206	55.4	125	78.4	413

Level of significance: * *p* ≤ 0.05; ** *p* ≤ 0.01, *** *p* ≤0.001; ns: not significant.
